# Effect of sarcopenia on survival in patients after pancreatic surgery: a systematic review and meta-analysis

**DOI:** 10.3389/fnut.2023.1315097

**Published:** 2024-01-08

**Authors:** Lei Zhong, Jifeng Liu, Mingquan Xia, Yunshu Zhang, Shuo Liu, Guang Tan

**Affiliations:** ^1^Department of General Surgery, The First Affiliated Hospital of Dalian Medical University, Dalian, China; ^2^Clinical Laboratory of Integrative Medicine, The First Affiliated Hospital of Dalian Medical University, Dalian, China; ^3^Department of Endocrinology and Metabolic Diseases, The First Affiliated Hospital of Dalian Medical University, Dalian, Liaoning, China

**Keywords:** sarcopenia, pancreatectomy, survival, cancer, meta-analysis

## Abstract

**Background:**

Numerous studies have reported sarcopenia to be associated with unfavorable outcomes in patients who have undergone pancreatectomy. Therefore, in this meta-analysis, we examined the relationship between sarcopenia and survival after pancreatic surgery.

**Methods:**

PubMed, Embase, and Cochrane Library were searched for studies that examined the association between sarcopenia and survival after pancreatic surgery from the inception of the database until June 1, 2023. Hazard ratio (HR) for overall survival (OS) and/or progression-free survival (PFS) of sarcopenia and pancreatic surgery were extracted from the selected studies and random or fixed-effect models were used to summarize the data according to the heterogeneity. Publication bias was assessed using Egger’s linear regression test and a funnel plot.

**Results:**

Sixteen studies met the inclusion criteria. For 13 aggregated univariate and 16 multivariate estimates, sarcopenia was associated with decreased OS (univariate analysis: HR 1.69, 95% CI 1.48–1.93; multivariate analysis: HR 1.69; 95% CI 1.39–2.05, I^2^ = 77.4%). Furthermore, sarcopenia was significantly associated with poor PFS of pancreatic resection (Change to univariate analysis: HR 1.74, 95% CI 1.47–2.05; multivariate analysis: HR 1.54; 95% CI 1.23–1.93, I^2^ = 63%).

**Conclusion:**

Sarcopenia may be a significant prognostic factor for a shortened survival following pancreatectomy since it is linked to an elevated risk of mortality. Further studies are required to understand how sarcopenia affects long-term results after pancreatic resection.

Systematic review registrationRegistration ID: CRD42023438208 https://www.crd.york.ac.uk/PROSPERO/#recordDetails.

## Introduction

Pancreatectomy is associated with good outcomes for numerous benign, premalignant, and malignant pancreatic tumors. However, it remains a challenging surgery with high morbidity due to postoperative complications and a low survival rate, particularly when performed for oncologic purposes (1, 2). These results can be attributed to the pancreatic gland texture (3), surgical nutritional support (4), the requirement for blood transfusions (5), and surgeon volume (6). Sarcopenia, meaning the degenerative loss of skeletal muscle mass, can be assessed through computed tomography (CT) measures of the psoas area and muscle density (7). Sarcopenia is linked to longer hospital stays, a higher risk of postoperative complications, and an increased risk of disability and recurrent hospitalization (8). Other detrimental effects of sarcopenia include mobility limitations, chronic illness, premature death, and frailty (9). Sarcopenia has recently been reported to possibly predict poor results in patients undergoing major abdominal surgeries (10–13). Despite some contradicting evidence, existing information on the relationship between sarcopenia and mortality in individuals after pancreatic surgery suggested that sarcopenia usually increases mortality in such patients (14–16). Moreover, a previously published International Study Group on Pancreatic Surgery Consensus Statement on Nutritional Support in Pancreatic Surgery reported that sarcopenia is a significant predictor of short-term and long-term outcomes and that long-term survival in patients with sarcopenia has consistently been low. However, the consensus statement is primarily based on observational research with a small sample size (17). Therefore, we conducted this systematic review and meta-analysis to further understand the effect of sarcopenia on patient survival following pancreatic resection.

## Methods

The study has been reported according to PRISMA (Preferred Reporting Items for Systematic Reviews and Meta-Analyses) and AMSTAR (Assessing the methodological quality of systematic reviews) guidelines (18, 19).

### Search strategy

PubMed, Embase, and the Cochrane Library databases were thoroughly searched for relevant papers released from the of date the database’s inception till June 1, 2023. The search phrases were (‘Sarcopenia’ OR ‘Muscular Atrophy’) AND (‘Pancreaticoduodenectomy’ OR ‘Pancreatectomy’ OR ‘Pancreaticojejunostomy’). Furthermore, all references in the eligible publications were carefully reviewed for new relevant studies. The search was conducted according to PRISMA Guidelines and included PICOs and cited references of PRISMA Guidelines (20).

### Selection criteria

Articles with data on the HR with a 95% confidence interval (CI) of sarcopenia and survival of pancreatic surgery were included. When the same data were used in two or more studies, only the most detailed study was selected. Studies not published in English and letters, case reports, reviews, expert comments, or editorials were excluded. Two researchers (LZ and SL) reviewed the titles and abstracts of all selected studies. Next, both researchers separately downloaded and rescreened the entire texts of any possibly relevant articles. Additionally, the reference lists of those papers were also screened for additional relevant articles that could be included.

### Data extraction

Every article was critically assessed by the two researchers (LZ and SL). For each article, we collected the following data: (1) study location, (2) sample size, (3) mean age of the sample, (4) sex ratio in every sample, (5) surgical procedures, (6) definitions of sarcopenia, (7) cut-off values of sarcopenia, (8) outcome of the research, including OS and/or PFS patients with sarcopenia who underwent pancreatic surgery, and (9) univariate and/or multivariate HR.

### Quality assessment

This meta-analysis utilized the Newcastle Ottawa scale (NOS) (21) to evaluate the quality of the included studies. The scale rates three categories, namely selection, comparability, and outcome, with a total of nine stars. An appropriate participant selection for the exposure and non-exposure cohorts was represented by 4 stars, while the comparability of the cohort was reflected by 2 stars. Lastly, three stars reflected the evaluation of the result and follow-up. Studies that scored >5 stars had moderate-to-high quality.

### Statistical analysis

Data on univariate and/or multivariate HR and 95% CI were extracted from the qualified studies and pooled to calculate an aggregating magnitude of effect by using fixed or random effect models according to the study’s heterogeneity. Univariate and multivariate HR were analyzed separately. For studies that provided multivariate analysis data, the HR with the most adjusted factors was used. The I^2^ statistic was applied to assess statistical heterogeneity between studies. The projected percentages of low, moderate, and high heterogeneity were 25, 50, and 75%, respectively. A sensitivity analysis was also performed to see if the excluded studies had a substantial influence on the result. When more than 10 original publications were included, publication bias was examined using funnel plotting and Egger’s test. All analyses were performed using STATA (version 16.0), and statistical significance was set at *p* < 0.05.

## Results

### Search results

**Figure 1:**
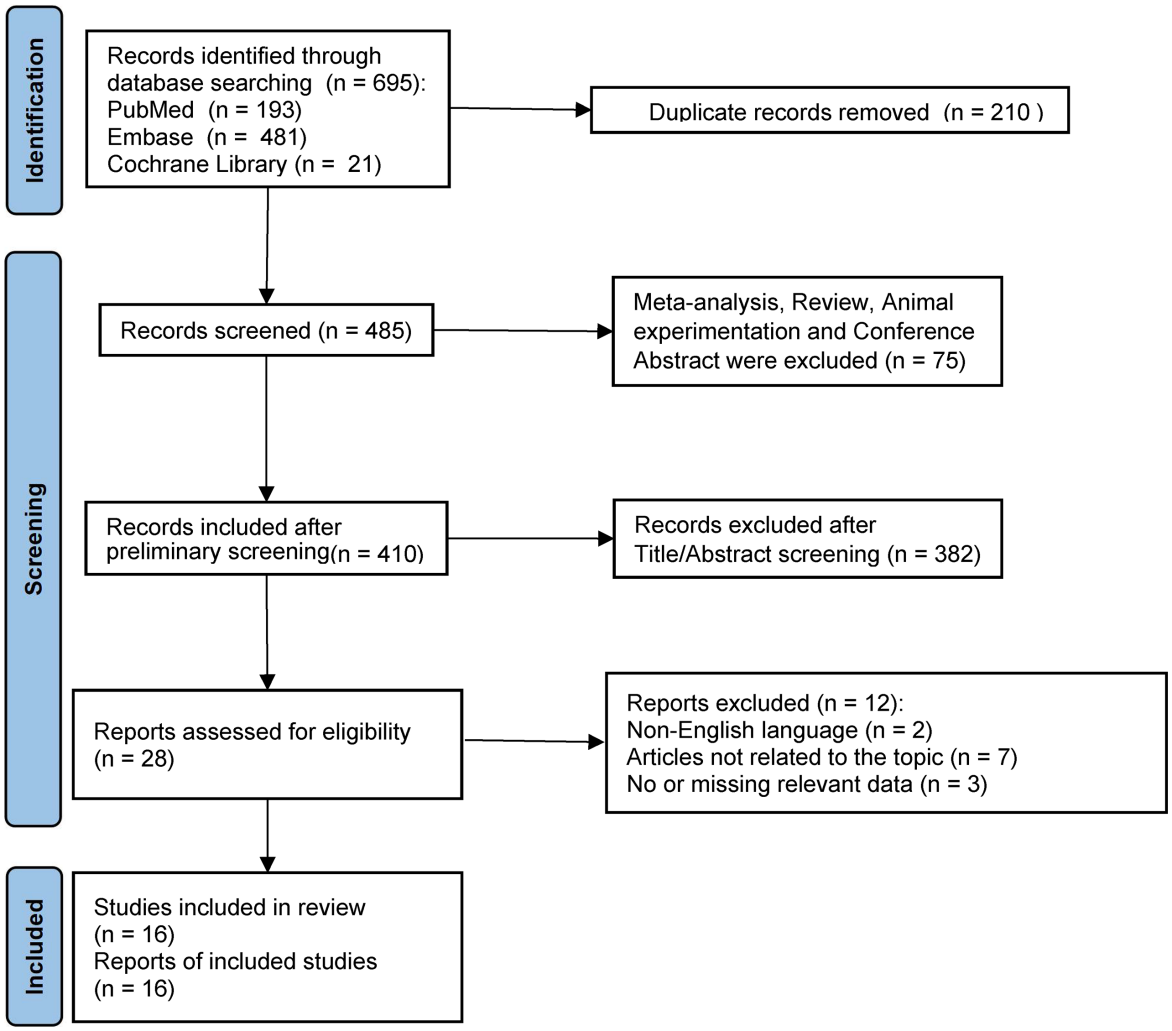
Process flow diagram for choosing studies for inclusion in the literature.

A flowchart depicting the literature screening procedure is shown in Figure 1. We identified 695 studies in the databases. After excluding studies that failed to meet the inclusion criteria and duplicate articles, 16 studies (13–16, 22–33) met the inclusion requirements for the analyses. A meta-analysis and systematic review were performed of these 16 studies.

### Study characteristics

All 16 included studies were c cohort analyses comprising a total of 4,250 patients included between 1996 and 2022. The sample sizes ranged from 83–763, and these studies had an average quality scoring of 7.4 stars. In total, multivariate OS results for 16 studies [13 studies (13–16, 22, 23, 25–31) were assessed for univariate estimates only] and multivariate PFS results for 6 studies (16, 23, 26, 30, 31, 33) were analyzed [five studies (16, 23, 26, 30, 31)were assessed for univariate estimates only]. The three main surgical procedures performed in the included studies were distal pancreatectomy (DP), total pancreatectomy (TP), and pancreaticoduodenectomy (PD). In addition to malignant tumors, such as pancreatic adenocarcinoma (PDAC) and periampullary cancer, benign pancreatic lesions like pancreatitis in the studies were also examined. Table 1 summarizes the basic characteristics of each included study. The sources of funding for each study included in the review are shown in Supplementary Table S1.

**Table 1:** Characteristics of the included studies.

Study	Country	Average age	Sample size	percentage of sarcopenic patients	Male/Female	Study interval	Disease	Types of resection	Overall survival (1,3,5 years)	Sarcopenia measure used and cut-off values	Study quality
Amini 2015	USA	67 (58–74)	763	152 (19.9%)	418/345	1996–2014	PDAC	PD, DP, TP	76.4, 34.9, 23.9%	TPV (cm^2^/m^2^) M: <17.2 *F* < 12.0	6★
Okumura 2015	Japan	67 (32–87)	230	64 (27.8%)	124/106	2004.1–2013.6	PDAC	PD, DP, TP, PPPD, SSPPD	–	PMI (cm^2^/m^2^) M: <5.9\u00B0F: <4.1	7★
Onesti 2016	USA	63.8 (18–93)	270	-	144/126	2005.7.1–2012.12.31	PDAC, DCC, Others	PD, DP, TP	–	LPMA (cm^2^) M: 920–1896\u00B0F: 601–1,131	8★
Ninomiya 2017	Japan	65.4 ± 10.1	265	170 (64.2%)	164/101	2005.5–2014.11	PDAC	PD, DP, TP	63.0, 18.9, 5.2%	SMI (cm^2^/m^2^) M: <43.75\u00B0F: <38.5	8★
Okumura 2017	Japan	68 (61–74)	301	120 (39.9%)	168/133	2004–2015	PC	PD, DP, TP	70.8, 21.9, 10.0%	SMI (cm^2^/m^2^) M: <47.1\u00B0F: <36.6	7★
Choi 2018	Korea	64.4 ± 9.3	180	60 (33.3%)	98/82	2008–2015	PC	PD, DP	67.3, 23.9, 16.0%	SMI (cm^2^/m^2^) M: <45.3 F: <39.3	8★
El Amrani 2018	France	61 ± 12	107	50 (47.6%)	51/56	2011.5–2015.7	PDAC, DCC, Others	PD, DP, TP	–	SMI (cm^2^/m^2^) M: <52.4 F: <38.5	6★
Sugimoto 2018	USA	65 (38–88)	323	200 (61.9%)	176/147	2000.3–2015.2	PDAC	PD, DP, TP	–	SMI (cm^2^/m^2^) M: <55.4 F: <38.9	8★
Gruber 2019	Austria	68 (34–87)	133	78 (58.6%)	68/65	2005–2010	PDAC	PD, DP	–	SMI (cm^2^/m^2^) M: <52.4 F: <38.5	7★
Ryu 2020	Korea	62.51 (24–88)	548	252 (46.0%)	326/222	2007.1–2016.6	PC	PPPD, PD, SSPPD	−, 26.0%	PMI (cm^2^/m^2^) M: <50.18\u00B0F: <38.63	8★
Peng 2021	China	66.2 ± 11.9	116	20 (17.2%)	68/48	2005.10–2018.8	PDAC	PD	56.0, 4.3, 0.0%	SMI (cm^2^/m^2^) M: <42.2 F: <33.9	7★
Aoki 2022	Japan	72.35 ± 8	83	14 (16.9%)	47/36	2016.1–2020.3	PC	–	–	SMI (kg/m^2^)M: <7 F: <6	7★
Kim 2022	Korea	63.6 ± 9.6	347	188 (54.2%)	202/145	2014.1–2017.1	PDAC	–	82.4, 45.8, 20.7%	SMI (cm^2^/m^2^) M: <42.2 F: <33.9	8★
Rom 2022	Israel	67 (61–75)	111	30 (27.0%)	59/52	2005–2017	PDAC	PD, DP	–	SMI (cm^2^/m^2^) M: <44.35\u00B0F: <34.82	7★
Shen 2023	China	59.9 ± 10.3	614	318 (61.6%)	368/246	2015.1–2022.5	PDAC	PD, DP, TP	–	SMI (cm^2^/m^2^) M: <52.4 F: <38.5	8★
Tazeoglu 2023	Turkey	60.45 ± 13.08	179	83 (46.3%)	105/74	2012.1–2022.1	PC	PD	–	PMI (cm^2^/m^2^) M: <5.3 F: <3.6	8★

PDAC, Pancreatic ductal adenocarcinoma; DCC, distal cholangiocarcinoma; PC, pancreatic cancer; Others: including ampullary cancer, solid-pseudopapillary tumor, chronic pancreatitis, intraductal papillary mucinous neoplasm, neuroendocrine tumor, gastrointestinal stromal tumor; PD, pancreatoduodenectomy; DP, Distal pancreatectomy; TP, total pancreatectomy; PPPD, pylorus-preserving PD, SSPPD, subtotal stomach-preserving PD; M, male; F, female; TPV, total psoas volume; PMI, psoas muscle mass index; LPMA, lean psoas muscle area; SMI, skeletal muscle mass index; −, not given or reported in the study.

### Meta-analysis results

#### Overall survival of patients after pancreatic surgery

**Figure 2:**
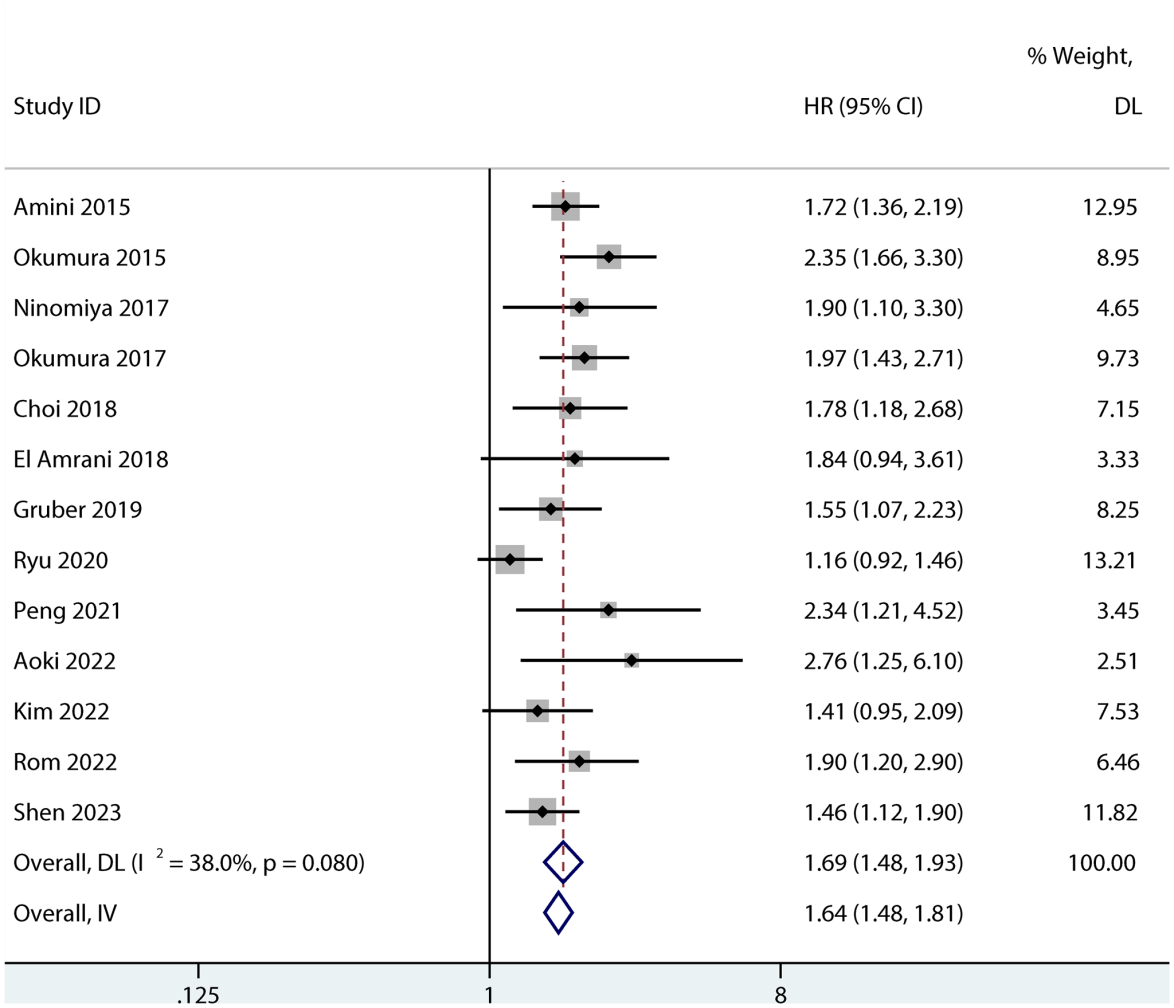
Forest plot of the univariate association between sarcopenia and OS for patients after pancreatic surgery.

**Figure 3:**
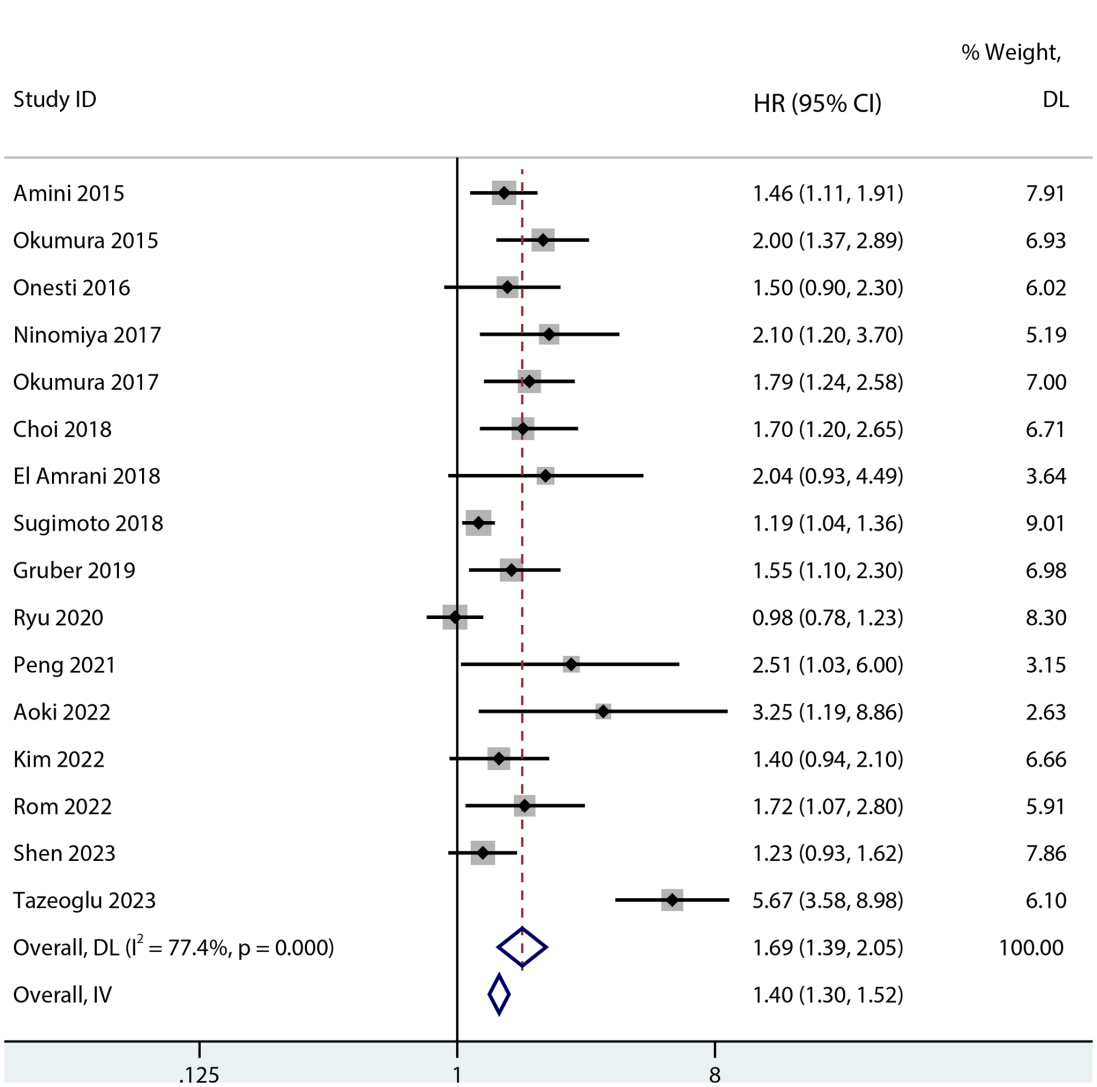
Forest plot of the multivariate association between sarcopenia and OS for patients after pancreatic surgery.

**Figure 4:**
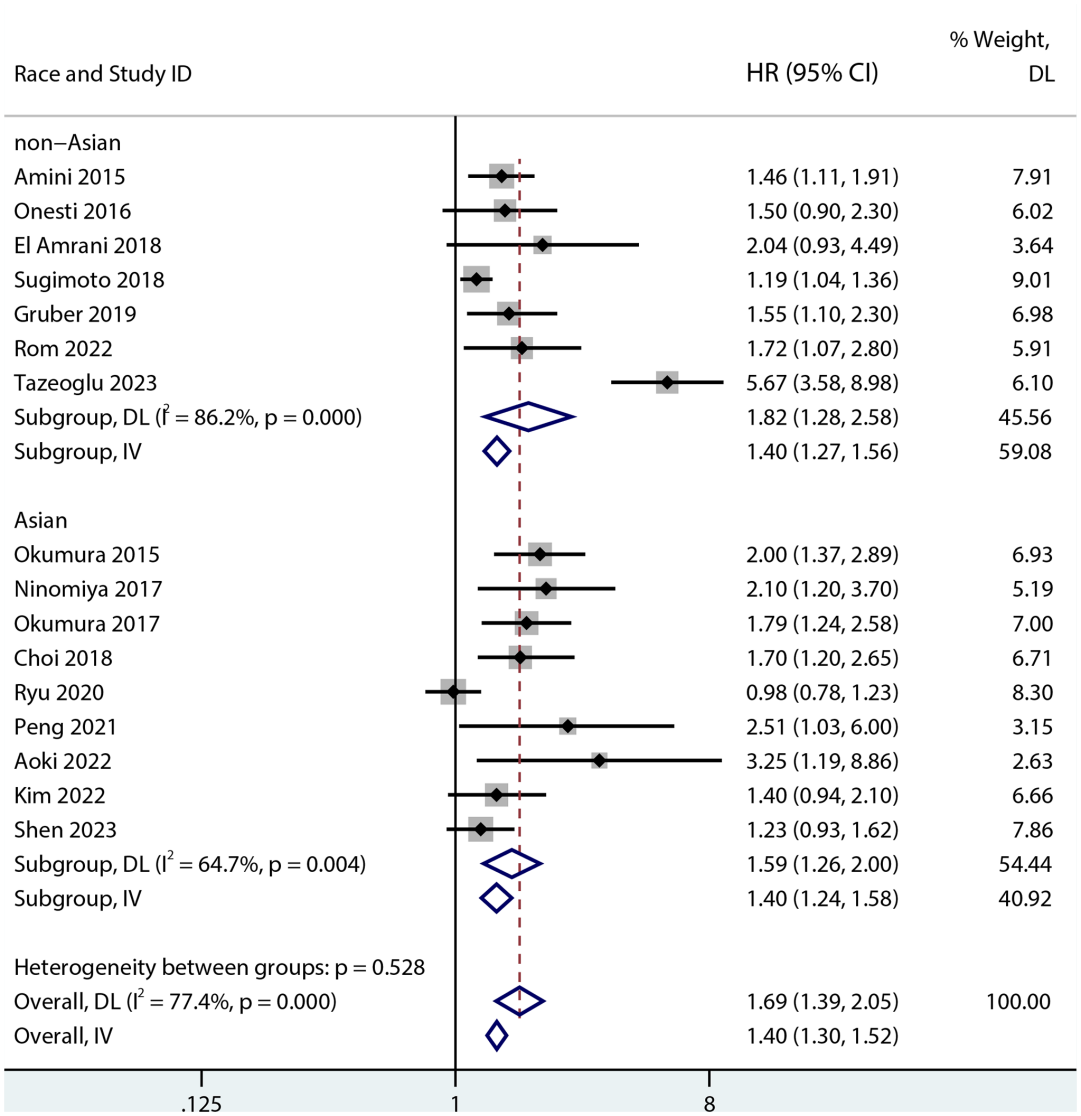
Forest plot of the association between sarcopenia and OS in both Asians and non-Asians after pancreatic surgery (multivariate analysis).

Sarcopenia was predictive of increased mortality risk among studies that provided a univariate HR (HR 1.69, 95% CI 1.48–1.93, *p* < 0.001, I^2^ = 38%; Figure 2). Additionally, sarcopenia was linked to a higher mortality risk according to the results of the aggregated multivariate HR (HR 1.69; 95% CI 1.39–2.05, I^2^ = 77.4%; Figure 3) analysis. To investigate potential causes of the high heterogeneity in the multivariate analyses, a subgroup analysis was performed. Both non-Asian (HR 1.82, 95% CI 1.28–2.58; Figure 4) and Asian (HR 1.59, 95% CI 1.26–2.00; Figure 4) studies demonstrated a pooled increased mortality risk associated with sarcopenia, and the impact of sarcopenia on OS after pancreatic surgery in non-Asians was more pronounced than in Asians.

#### Progression-free survival of patients with pancreatic cancer after pancreatic surgery

**Figure 5:**
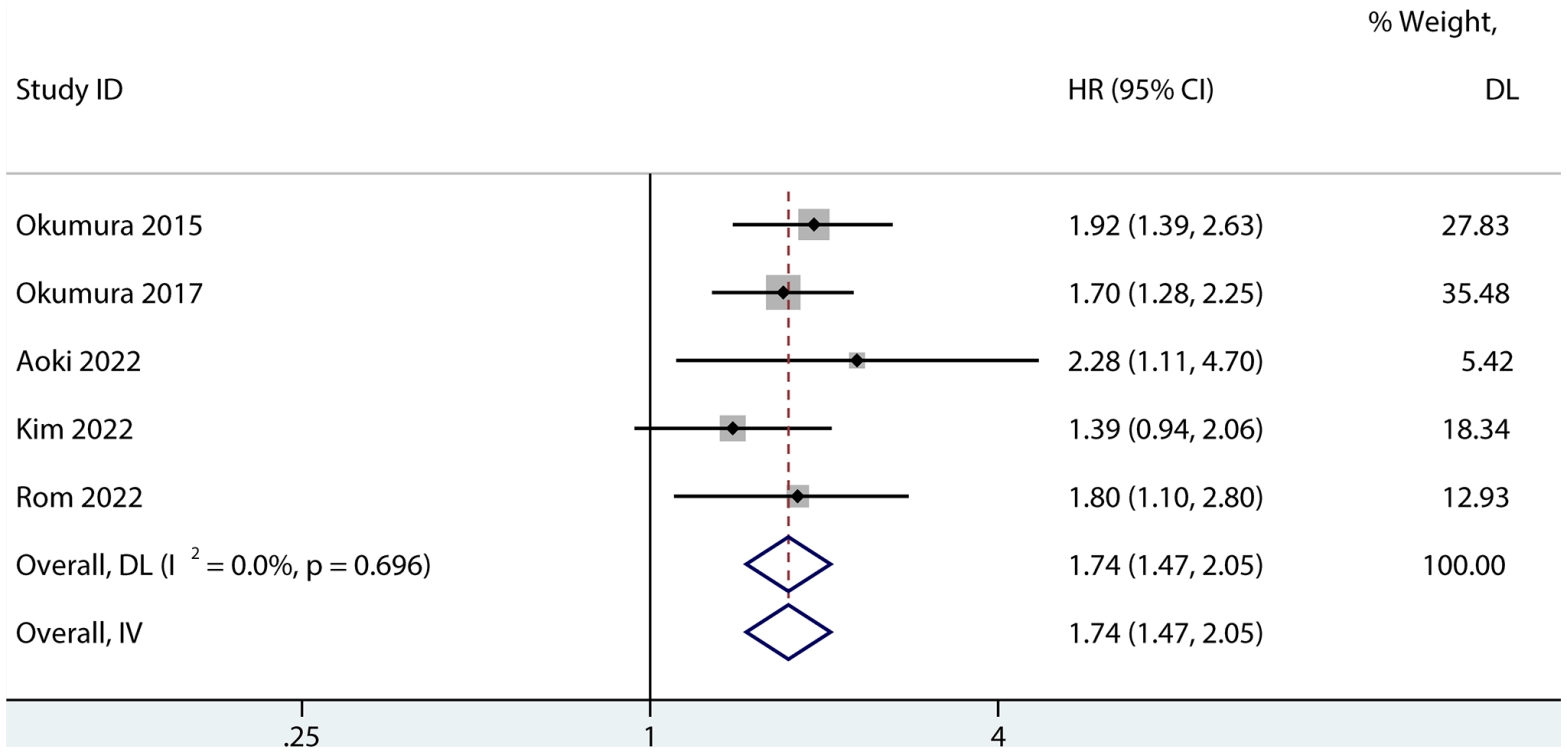
Forest plot of the univariate association between sarcopenia and PFS for patients with pancreatic cancer after pancreatic surgery.

**Figure 6:**
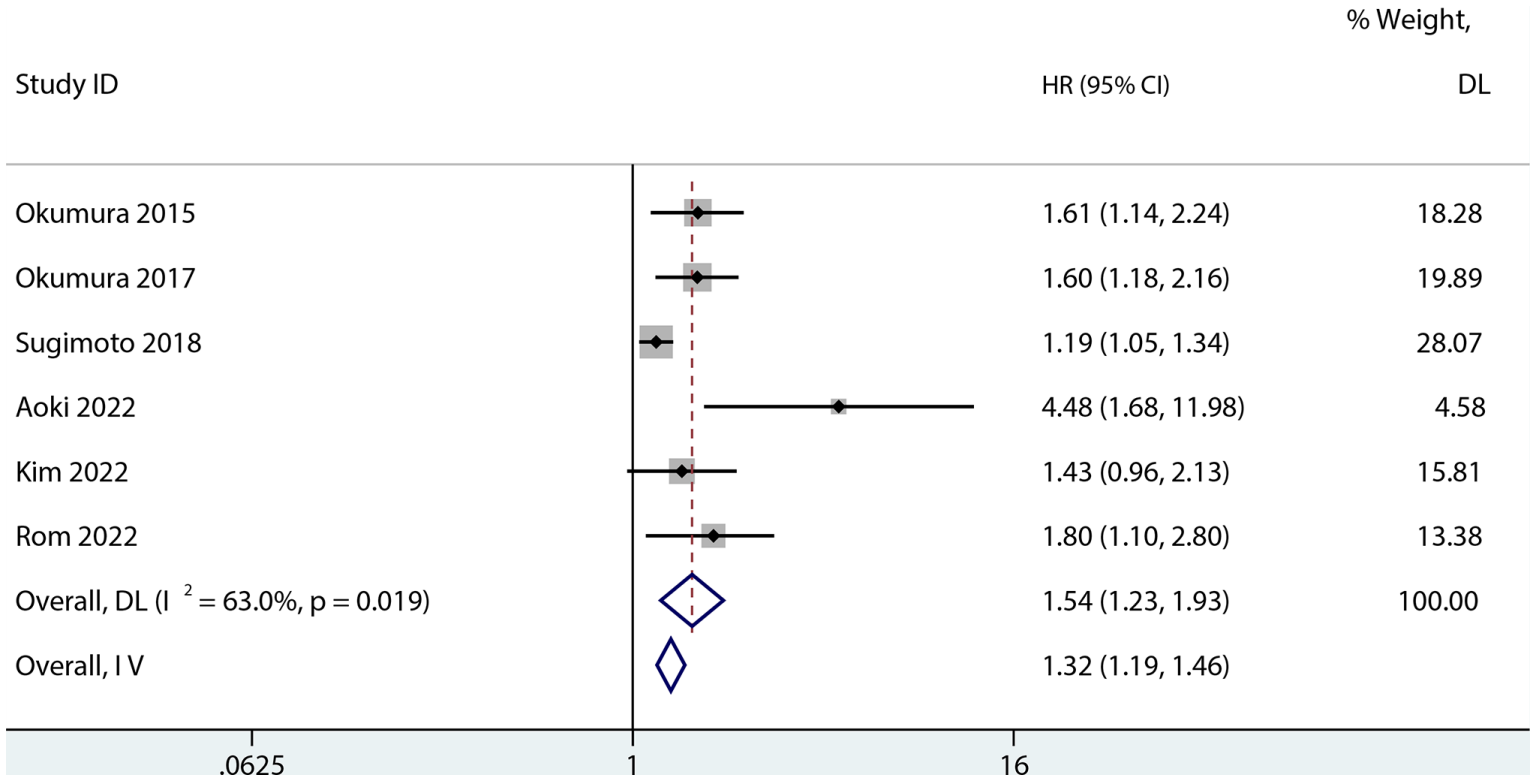
Forest plot of the multivariate association between sarcopenia and PFS for patients with pancreatic cancer after pancreatic surgery.

Six studies assessed the relationship between PFS and sarcopenia after pancreatic cancer surgery. Among the studies that reported a univariate HR, the results suggested that sarcopenia reduces PFS in patients after pancreatectomy (HR 1.74; 95% CI 1.47–2.05, *p* < 0.001, I^2^ = 0.0%; Figure 5). Sarcopenia also had a negative influence on PFS after pancreatic surgery even after risk factor adjustment (1.54; 95% CI 1.23–1.93, I^2^ = 63%; Figure 6). However, no subgroup analysis for PFS was done due to the small number of primary studies.

#### Overall survival of patients with pancreatic adenocarcinoma after pancreatic surgery

**Figure 7:**
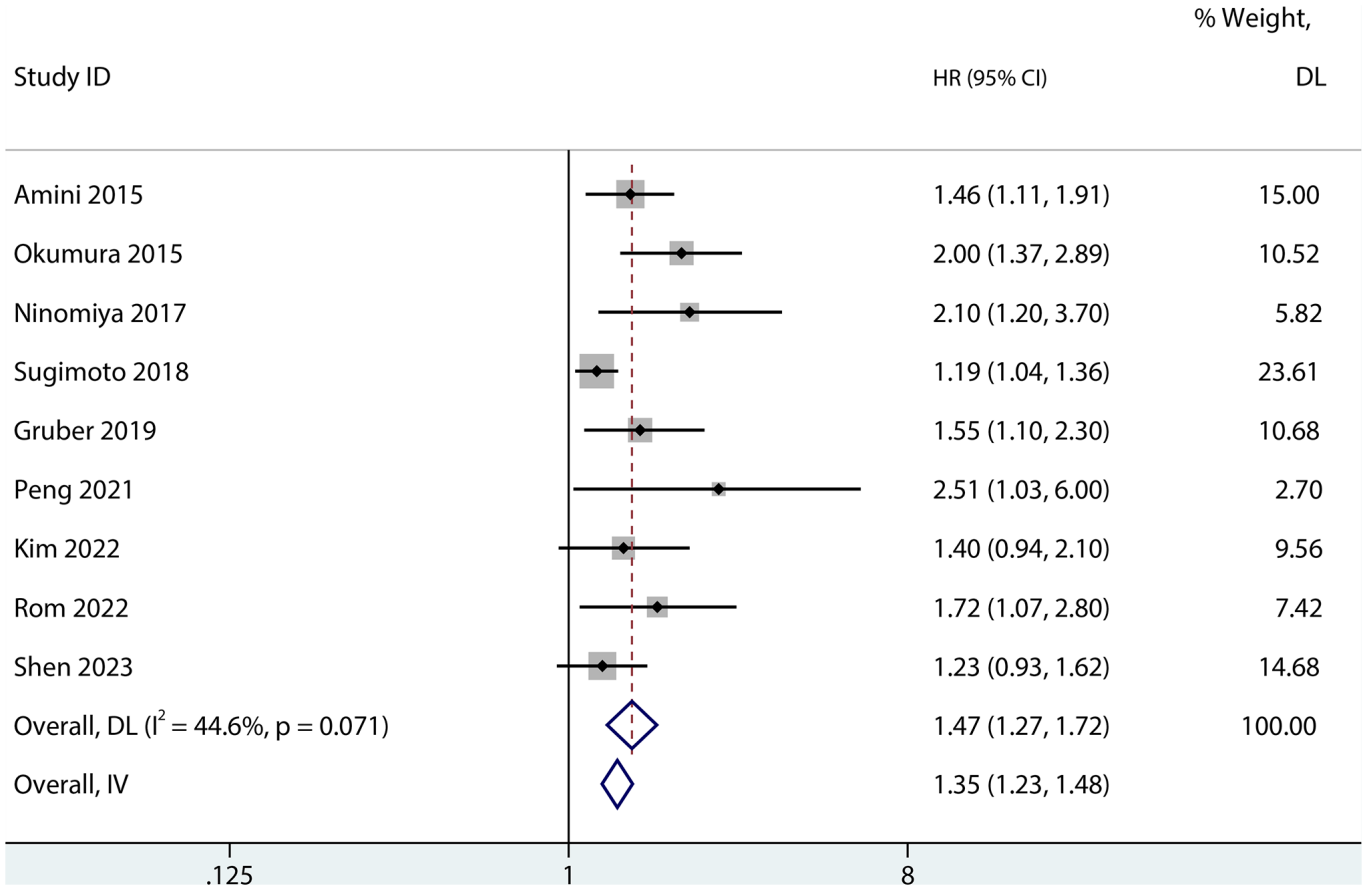
Forest plot of the association between OS in patients with PDAC after pancreatic surgery.

The influence of sarcopenia on the postoperative OS of patients with PDAC was reported in nine studies (13, 22, 23, 25, 28–31, 33). The aggregated multivariate HR analysis indicated that sarcopenia was associated with lower postoperative OS of patients with PDAC (HR 1.47; 95% CI 1.27–1.72, I^2^ = 44.6%; Figure 7).

#### Sensitivity analysis and publication bias

Sensitivity analyses of studies that conducted univariate and multivariate analyses of the effect of sarcopenia on OS showed that arbitrary deletion of studies would not affect the results of this meta-analysis, indicating stable and reliable results (Supplementary Figures S1, S2). Either Egger’s regression analysis (*p* > 0.05) or funnel plots (Supplementary Figures S3, S4) indicated the presence of possible publication bias for the univariate and multivariate analyses.

## Discussion

Despite advancements in perioperative care and surgical techniques improving pancreatic surgery results, surgical morbidity and death are significant concerns (34, 35). Sarcopenia, which is traditionally characterized as decreased lean skeletal muscle mass coupled with impaired nutritional status and immune function, is an integrated, quantitative indication of body reserve (36–38). Recently research has shown that preoperative sarcopenia is linked to a worse OS for patients with PDAC (39–41). However, the effects of sarcopenia on patient survival following pancreatic surgery remain debatable. This is the first meta-analysis to investigate the predictive value of sarcopenia on post-pancreatectomy patient survival. Of note, this study included both benign and malignant pancreatic diseases, thereby incorporating all possible reasons for pancreatic resection. Consistent with most previous study findings (13, 32), the present meta-analysis demonstrates that sarcopenia was associated with a poor prognosis in patients who underwent pancreatic surgery. Patients with sarcopenia have significantly shorter long-term OS in univariate or multivariate analyses than those without sarcopenia. However, the multivariate meta-analysis evaluating the impact of sarcopenia on OS following pancreatic surgery revealed significant heterogeneity. The subgroup analysis identified the inclusion of various racial groups in the different studies as a possible source of heterogeneity and that sarcopenia had a more negative effect on OS in non-Asians following pancreatic surgery than in Asians. This is also the first meta-analysis to examine the impact of sarcopenia on the postoperative survival rate in patients undergoing pancreatic surgery in different populations. Postoperatively, in patients with pancreatic cancer, sarcopenia was associated with a reduced postoperative PFS. Moreover, postoperatively, in patients with PDAC, sarcopenia was linked to a poorer OS according to a meta-analysis of the results from nine trials.

Several discrepancies have persisted between the definition and diagnostic criteria of sarcopenia in the last dozen years. Most original sarcopenia working groups in their definition and diagnosis criteria have defined sarcopenia as reduced muscle mass (42, 43). It was only later that researchers reported that muscle strength function could better predict sarcopenia and suggested adding them to the definition and diagnostic criteria of sarcopenia (44, 45). In the latest Sarcopenia Definitions and Outcomes Consortium diagnostic criteria, muscle mass has been removed, and instead, sarcopenia has been defined by muscle strength and function (46). The prevalence of sarcopenia varies widely across most meta-analyses, despite recent studies using relatively consistent definitions (47, 48). This is because the use of different cut-offs and measurements reveals different prevalence results. Although the studies included in our meta-analysis all used imaging methods to assess muscle mass, they used different cut-off values, possibly affecting the final analysis results.

In most studies, preoperative or baseline sarcopenia was linked to a higher risk of postoperative infection, longer hospital stays, and an elevated risk of short-and long-term death (49–52). Besides, the risks differed for various patient groups in terms of survival, death rates, and other unfavorable outcomes. Patients who had emergency laparotomies had the highest all-cause mortality (52), whereas those who underwent radical cystectomies had the lowest (51). These differences may be largely due to the different definitions of sarcopenia used and may also be influenced by different measurement methods for muscle mass, strength, and function. MRI and CT scans are the gold standard for the non-invasive assessment of muscle mass (53); nevertheless, their use in primary care and research is limited due to cost, accessibility, absence of portable equipment, and the need for highly skilled people (54). Clinically, grip strength is the most widely used, economical, and straightforward metric for assessing muscular strength (55), and gait speed is the most utilized tool for evaluating muscle function (56). Low grip strength and gait speed are good predictors of adverse outcomes, such as longer hospital stays, poor health-related quality of life, and all-cause mortality (57, 58). Hence, compared with CT or MRI, grip strength and gait speed are easier to measure. Additionally, similar to decreased muscle mass, decreased muscle strength and function are risk factors for adverse outcomes in several diseases. All studies included in our analysis used the criteria of low muscle mass to assess sarcopenia, which may reduce the comprehensiveness of our study.

Sarcopenia results in muscle weakness, decreased muscle mass, and impaired muscle function. These factors may raise the surgical risk, such as the difficulty of the surgical procedure, the higher prevalence of postoperative complications, and make it more difficult for patients to recover postoperatively, which negatively affected patient survival (25, 59, 60). However, Chathura et al. (61) discovered that preoperative sarcopenia was not linked to a higher incidence of any particular postoperative complication. Aoki et al. performed a multivariate analysis and reported sarcopenia as the most significant risk factor for poor RFS and OS (16). Notably, muscle mass loss may lead to frailty. Although there are many similarities between the physical signs of sarcopenia and frailty, frailty as a complicated geriatric syndrome that covers a wider range of geriatric decline, including cognitive and social impairment linked to negative outcomes (62). When compared to non-frail patients, the preoperative presence of frailty was linked to a threefold increase in long-term mortality, a sixfold increase in the risk of early postoperative mortality, and a twofold increase in the chance of developing significant postoperative morbidity (63). Therefore, for patients with sarcopenia, a comprehensive assessment of the patient’s physical condition preoperatively is necessary to increase the therapeutic effect of the surgery.

As we all know that poor prognostic factors for post-pancreatic surgery mainly included large tumor size, higher levels of CA 19–9, nodal involvement, involved resection margins, TNM stage, and the need for neoadjuvant chemotherapy (64). Rom et al. (31) reported that sarcopenia was associated not only with the above adverse prognostic factors but also with poor survival after pancreatic surgery, supporting our findings that sarcopenia is an early surrogate radiological marker of aggressive tumor biology that predicts a poor prognosis. Furthermore, a recent study has reported that sarcopenia with differentiated degrees of PDAC has different prognostic values (13). Shen et al. speculated that this may be because tumor-associated sarcopenia is more severe in poorly differentiated PDAC than in moderate or highly differentiated PDAC and sarcopenia can be largely reversed in patients with moderate or highly differentiated PDAC but not in patients with poorly differentiated PDAC (13). However, more prospective studies are needed to confirm this.

Of note, postoperative radiotherapy and chemotherapy are routinely required for pancreatic malignancies (64). A recent study on the relationship between sarcopenia and prognosis after surgery for gastrointestinal cancer showed that there were significantly more patients undergoing postoperative chemotherapy radiation in the sarcopenic group than in the nonsarcopenic group and that patients with sarcopenia had significantly more chemotherapy changes, including delays, dose reduction, or termination (65). These findings indicate that following abdominal surgery for digestive tract cancer, sarcopenia had a negative impact on chemotherapy and radiation, particularly on the former. Interestingly, recent studies have also reported a significant reduction in skeletal muscle mass during chemotherapy (66), and sarcopenia is associated with major chemotherapy toxicities (such as diarrhea, infection, alopecia and neuro-pathy) (67, 68). Consequently, in light of the earlier discoveries, it is proposed that sarcopenia detection before, during, and following chemotherapy is crucial for focused nutritional intervention that is intended to enhance the results of chemotherapy treatment.

The mechanisms through which sarcopenia increases the risk of tumor recurrence and death remain unknown. However, it might be connected to the following factors. First, people with sarcopenia may have a poorer tolerance for chemotherapy according to a study that reported that sarcopenia is linked to lower chemotherapy tolerance in individuals with different cancers (69–71). Given that adjuvant chemotherapy is a significant independent protective factor for both OS and disease-free survival, lower chemotherapy tolerance may contribute to sarcopenia’s detrimental effect on long-term survival. Second, sarcopenia may be a reflection of high metabolic activity due to a more aggressive tumor biology, leading to more serious systemic inflammation and, consequently, muscle wasting (72).

Sarcopenia can also affect patients with benign disease. Brittany et al. (73) reported an increased incidence of overall as well as major complications among patients with sarcopenia among individuals without a cancer diagnosis. One explanation is that major surgery is known to be associated with biochemical cytokine response, causing persistent inflammation and immunosuppression, leading to prolonged severe illness and poor survival (74). Moreover, patients with sarcopenic patients may be more “vulnerable” to negative outcomes as an impact of this reaction (75). However, to fully understand the association between sarcopenia and postoperative survival in non-tumor patients, further research is required.

To lower the risks, perioperative therapies are crucial. The complex etiology of sarcopenia significantly influences effective prevention and treatment of isolated drugs and/or nutritional strategies (76). To manage sarcopenia more effectively, multimodal solutions, such as a combination of exercise regimens and dietary treatments, need to be developed (77). Some studies have indicated that certain dietary patterns, such as sufficient protein, vitamin D, antioxidant elements, and long-chain polyunsaturated fatty acid intake, can help control sarcopenia (78). Additionally, a high total protein diet can also protect against frailty (79). In individuals who are already frail, protein-energy supplementation may reduce the progression of functional decline (80). Nevertheless, currently, no solid recommendations on nutrition therapy for sarcopenia and frailty exist because of heterogeneous data and a lack of major clinical trials (81, 82). Exercise therapies, particularly those based on resistance training, may be able to enhance athletic ability and increase muscle mass and power (83). Additionally, resistance training prevents sarcopenia development in the most cost-efficient manner and enhances multiple facets of overall wellness (84). Patients with pancreatic cancer might benefit from supportive treatment that emphasizes diet and exercise because malnutrition is a common occurrence in this population (85). Such supportive therapy would be best administered during neoadjuvant chemoradiation therapy, as patients undergoing these treatments are more likely to experience steatotic changes and lose muscle mass due to reduced oral intake and physical activity (26).

This study had some limitations. First, a subgroup analysis to assess how different definitions and cut-off values affected the final results could not be performed because the definitions of sarcopenia in the included studies and their cut-off values varied. Moreover, because the included study did not use muscle strength and function to assess sarcopenia, we could not comprehensively analyze the effects of sarcopenia and pancreatic postoperative prognosis; thus, future studies are required to explore this. Second, the analysis could have decreased credibility because of insufficient research on sarcopenia and PFS following pancreatic surgery. Third, the adjusted HR offered estimates after accounting for any confounding risk variables; however, most studies did not account for the same risk factors, such as changes in patient profiles, improvements in the perioperative patterns of care, and the balance between the safety and efficacy of adjuvant therapy, which might impact the final outcomes of this analysis. Fourth, due to insufficient data from the original study, no subgroup analysis was performed on tumor and non-tumor patients, which may have had some impact on the final results. Lastly, publication bias may be present, and research with no substantial impacts may not have been published. Hence, the given data may overestimate the genuine effect. Moreover, our study did not include studies that defined sarcopenia in terms of muscle strength and function, which may also have contributed to publication bias. Only English studies were included, possibly leading to selection bias. Therefore, we still need a large number of multicenter, prospective studies to verify the association between sarcopenia and survival after pancreatic surgery.

In summary, sarcopenia is related to poor OS after pancreatic surgery. Moreover, it can reduce the PFS in patients with pancreatic cancer after pancreatectomy. Moreover, for patients with PDAC, sarcopenia can negatively affect their OS postoperatively. More research is needed to validate our findings, and the causes underlying malnutrition in this population undergoing pancreatic surgery must be understood and improved in future studies.

## Data availability statement

The original contributions presented in the study are included in the article/Supplementary material, further inquiries can be directed to the corresponding authors.

## Author contributions

LZ: Data curation, Investigation, Methodology, Software, Writing – review & editing. JL: Writing – review & editing. SL: Writing – original draft. GT: Writing – review & editing. MX: Data curation, Methodology, Writing – review & editing. YZ: Writing – review & editing.

## Supplementary material

The Supplementary material for this article can be found online at:

https://www.frontiersin.org/articles/10.3389/fnut.2023.1315097/full#supplementary-material
