# Supplementary material for: Effect of sarcopenia on survival in patients after pancreatic surgery: a systematic review and meta-analysis
Source: Front Nutr. 2024 Jan 8;10:1315097. doi: 10.3389/fnut.2023.1315097 (PMC10800600; doi:10.3389/fnut.2023.1315097)
Supplement: Supplementary file 1 [file Table_1.docx]

Table S1. Sources of funding for individual studies included in the review.

| Study | Sources of financial support |
| --- | --- |
| Amini 2015 | None |
| Okumura 2015 | None |
| Onesti 2016 | None |
| Ninomiya 2017 | None |
| Okumura 2017 | None |
| Choi 2018 | National Research Foundation of Korea (grant number NRF-2015R1C1A1A02037568) |
| El Amrani 2018 | None |
| Sugimoto 2018 | This study was supported in part by a NIH grant P50CA102701, Mayo Clinic SPORE in Pancreatic Cancer |
| Gruber 2019 | None |
| Ryu 2020 | None |
| Peng 2021 | National Taiwan University Hospital and Industrial Technology Research Institute cooperation research project (PC1233) |
| Aoki 2022 | None |
| Kim 2022 | Ministry of Health & Welfare, Republic of Korea (grant number: HI18C1216) |
| Rom 2022 | None |
| Shen 2023 | National Natural Science Foundation of China (82002579) |
| Tazeoglu 2023 | None |
