# Supplementary figures and images for: Effect of sarcopenia on survival in patients after pancreatic surgery: a systematic review and meta-analysis
Source: Front Nutr. 2024 Jan 8;10:1315097. doi: 10.3389/fnut.2023.1315097 (PMC10800600; doi:10.3389/fnut.2023.1315097)

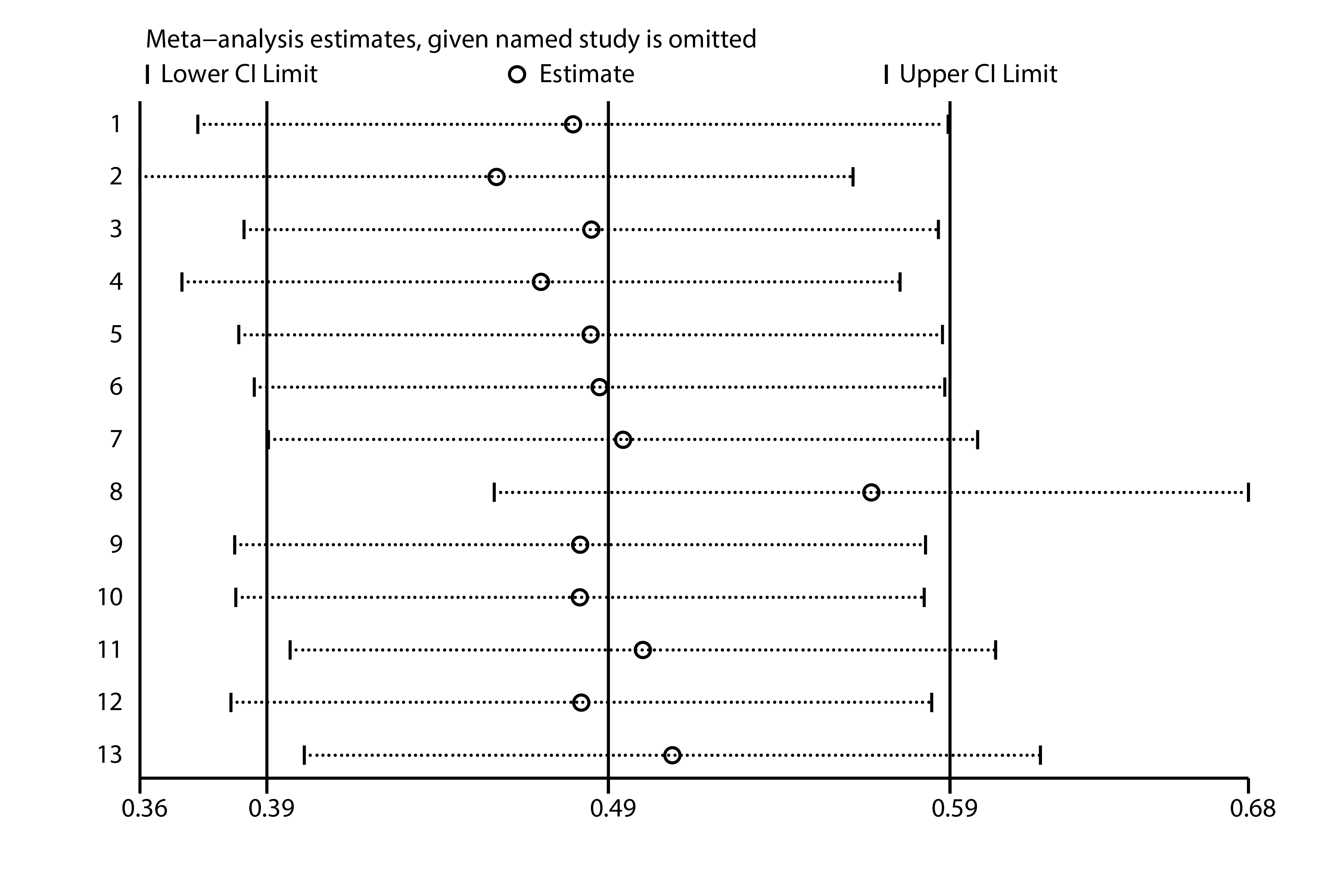

Supplement: Supplementary file 2 [file Image_1.TIF]

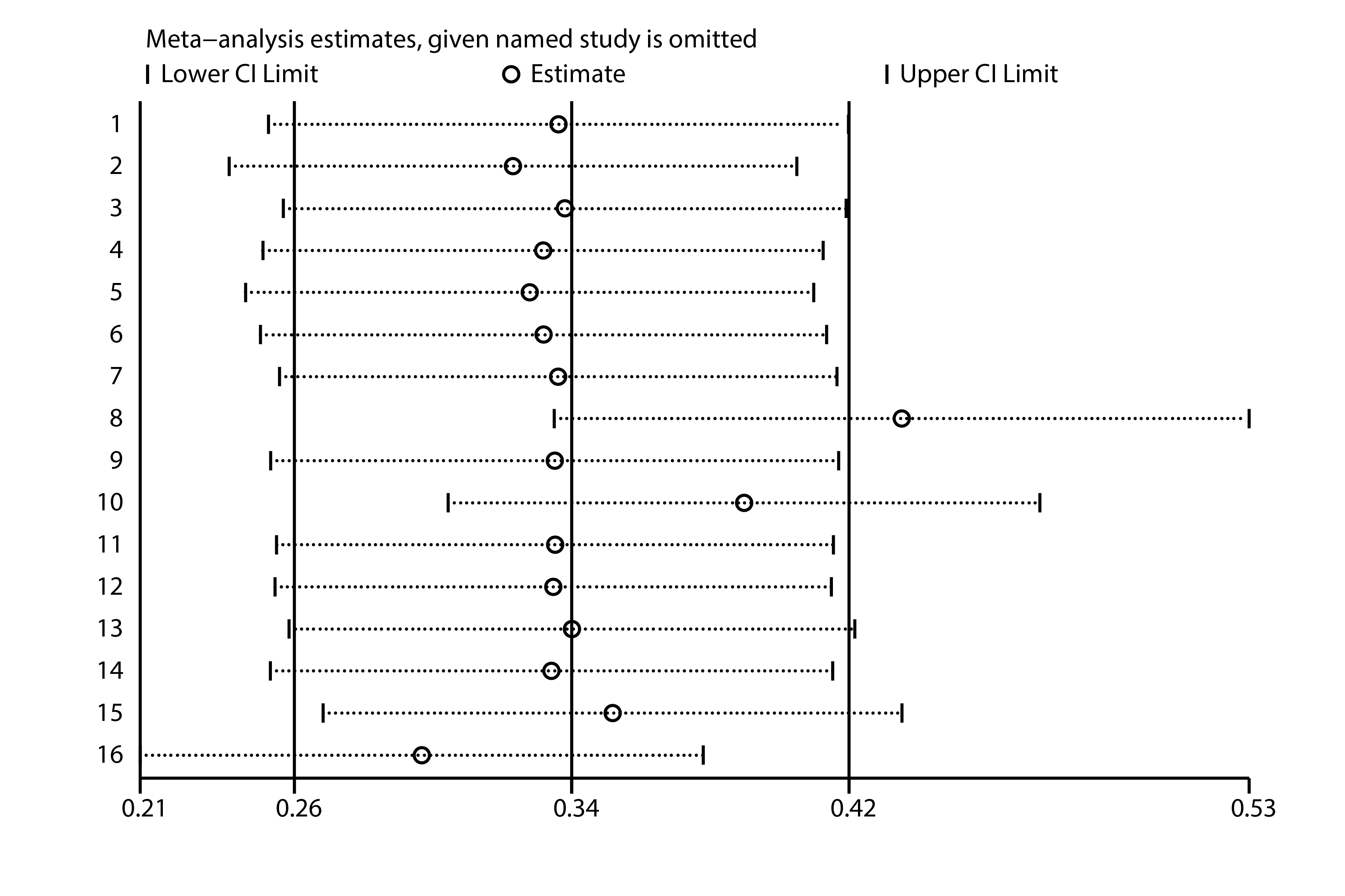

Supplement: Supplementary file 3 [file Image_2.TIF]

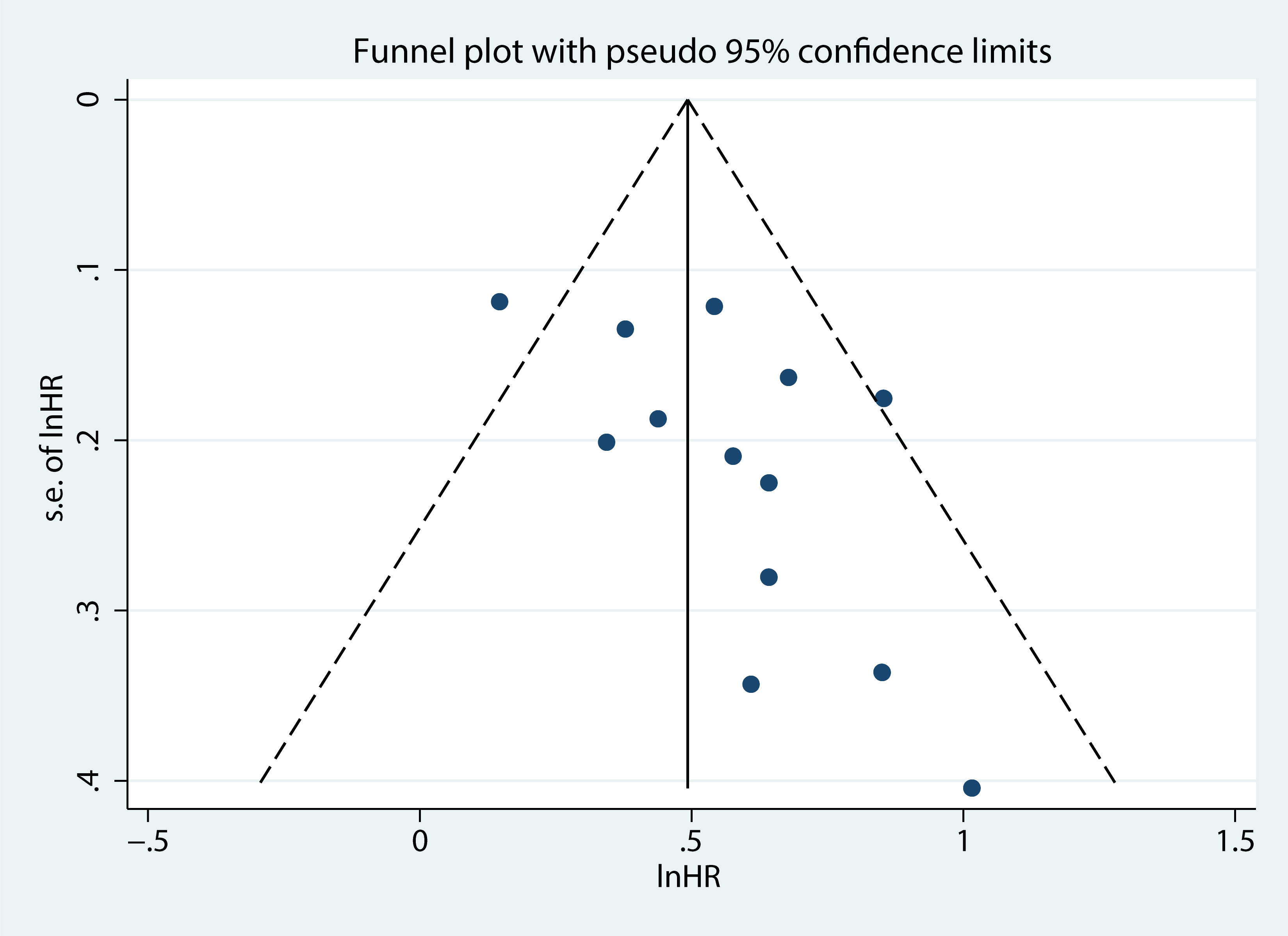

Supplement: Supplementary file 4 [file Image_3.TIF]

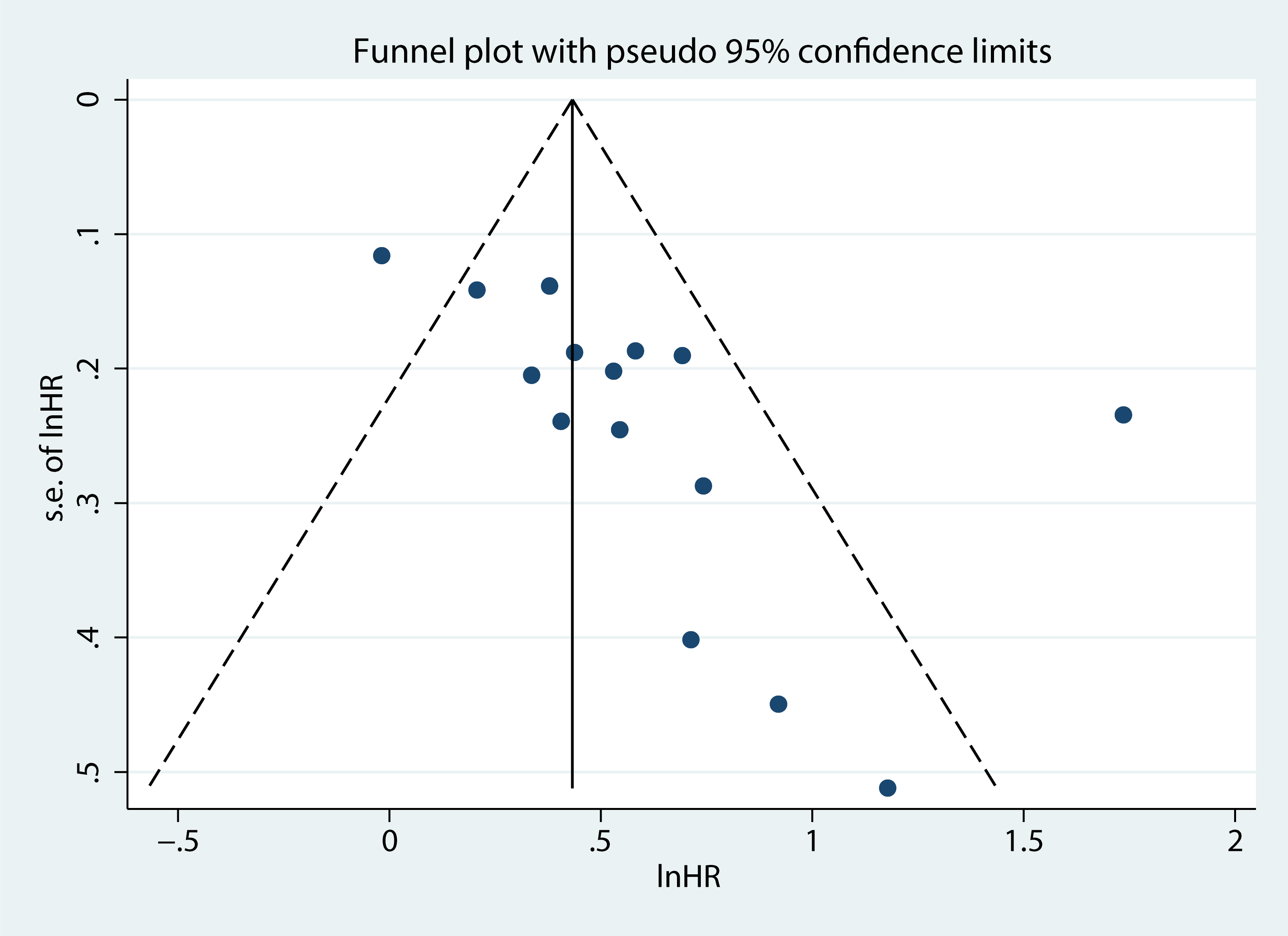

Supplement: Supplementary file 5 [file Image_4.TIF]
